# Dental Pulp Polyps Contain Stem Cells Comparable to the Normal Dental Pulps

**DOI:** 10.4317/jced.51305

**Published:** 2014-02-01

**Authors:** Armin Attar, Mohamadreza B. Eslaminejad, Maryam S. Tavangar, Razieh Karamzadeh, Ali Dehghani-Nazhvani, Yasamin Ghahramani, Fariba Malekmohammadi, Seyed M. Hosseini

**Affiliations:** 1 Cellular and Molecular Research Club, Shiraz University of Medical Sciences, Shiraz, Iran; 2 Department Cardiovascular Medicine, Shiraz University of Medical Sciences, Shiraz, Iran; 3 Student research committee, School of Medicine, Shiraz University of Medical Sciences, Shiraz, Iran; 4 Department of Stem Cells and Developmental Biology at Cell Science Research Center, Royan Institute for Stem Cell Biology and Technology, ACECR, Tehran, Iran; 5 Department of Operative Dentistry, Dental Faculty, Shiraz University of Medical Sciences, Shiraz, Iran; 6 Biomaterial Research center, Dental Faculty, Shiraz University of Medical Sciences, Shiraz, Iran; 7 Department of Oral Pathology, Dental Faculty, Shiraz University of Medical Sciences, Shiraz, Iran; 8 Department of Endodontics, Dental Faculty, Shiraz University of Medical Sciences, Shiraz, Iran

## Abstract

Objectives: Few studies investigated the isolation of stem cells from pathologically injured dental tissues. The aim of this study was to assess the possibility of isolation of stem cells from pulp polyps (chronic hyperplastic pulpitis), a pathological tissue produced in an inflammatory proliferative response within a tooth. Study design: Pulp polyp tissues were enzymatically digested and the harvested single cells were cultured. Cultured cells underwent differentiation to adipocytes and osteoblasts as well as flowcytometric analysis for markers such as: CD90, CD73, CD105, CD45, and CD14. In addition we tried to compare other characteristics (including colonigenic efficacy, population doubling time and the cell surface antigen panels) of these cells to that of healthy dental pulp stem cells (DPSCs). Results: Cells isolated from pulp polyps displayed spindle shape morphology and differentiated into adipocytes and osteoblasts successfully. These cells expressed CD90, CD73, and CD105 while were negative for CD45, CD14. Number of colonies among 104 tissue cells was higher in the normal pulp tissue derived cells than the pulp polyps (P=0.016); but as polyp tissues are larger and contain more cells (P=0.004), the total number of the stem cell in a sample tissue was higher in polyps but not significantly (P=0.073). Conclusions: The cells isolated from pulp polyps fulfill minimal criteria needed for MSC definition; hence, it can be concluded that pulp polyps contain stem cells. Although pulp polyps are rare tissues in daily practice but when they are present, may serve as a possible new non-invasively acquired tissue resource of stem cells for affected patients. List of abbreviations: APC = allophycocyanin, BM = Bone Marrow, CFU-F = Colony Forming Unit Fibroblast, DPSC = Dental Pulp Stem Cell, FITC = fluorescein isothiocyanate, MNC = mononuclear cells, MSC = Multipotent Mesenchymal Stromal Cell, PE = Phycoerythrin, PerCP = Peridinin chlorophyll protein, PPSC = Pulp Polyp Stem Cell.

** Key words:**Adult stem cell, chronic hyperplastic pulpitis, dental pulp stem cell, pulp polyp.

## Introduction

Multipotent mesenchymal stromal cells (MSCs)”, previously known as “mesenchymal stem cells” (1), are clonogenic, plastic adherent cells with multiple differentiation capacity into mesenchyme and even non-mesenchyme lineage cells such as adipocyte, osteoblast, chondrocyte, hepatocyte and neural cell (2). The ordinary resource of MSCs is bone marrow (BM), while other sources like adipose tissue (3), umbilical cord (4) and also dental pulp (5) are considered as suitable candidates.

Dental pulp is an ‘ecto-mesenchyme’ derived tissue as it has originated from the earlier interaction of mesenchyme with the neural crest. Although dental pulp stem cells (DPSCs) share common features with BM-MSCs, they may be more committed to odontogenic rather than osteogenic development (6). Several attempts have been made to isolate stem cells from dental tissues other than adult pulp, including deciduous teeth (7), periodontal ligament (8), dental follicle (9) and apical papilla (10). But only few studies have been done on evaluating the presence of stem cells in dental tissues affected by a pathological process (11,12). All these studies evaluated the presence of stem cells in the normal tissues affected by inflammation. We aimed to evaluate the presence of stem cells within a tissue that is fully formed from a pathologic process, pulp polyps. The pulp polyp, also known as chronic hyperplastic pulpitis or proliferative pulpitis, is a type of inflammatory hyperplasia. It occurs in a vital tooth with a good blood supply when the pulp has been exposed to caries or trauma (13). Here, there was an attempt to assess the possibility of isolation of stem cells from pulp polyps and compare the characteristics of isolated cells with that of DPSCs.

## Material and Methods

1. Preparation of single cell suspension from pulp polyps

Eight pulp polyp samples were collected from permanent molar teeth. Based on the current definition (13), the diagnosis of chronic hyperplastic pulpitis was done by endodontics specialists. All the patients were adolescents with a history of untreated carious lesions but without spontaneous prolonged pain. The teeth responded to the electrical pulp testing. No internal resorption or periapical periodontitis were observed on radiographs. All the patients gave their written informed consent before enrollment in the study. This study conformed to the declaration of Helsinki and was approved by the local Ethics Committee.

Polyp tissues were removed from the pulp chamber through curettage. The samples were transferred in PBS-EDTA solution with 1% penicillin/streptomycin and 1% Fungizone (both from Gibco/ Invitrogen, Carlsbad, CA, USA). The tissues were minced in sterile condition, undergoing enzymatic digestion with a solution of collagenase type I 3mg/ml and dispase type II 4mg/ml (both from Sigma, St. Louis, MO, USA) for 1 hr with occasional shaking. The obtained single cell suspension was passed through 70μm cell strainer (BD Biosciences, San Jose, CA, USA) and centrifuged with 300g for 10 min to remove the enzymes. The cells were then resuspended in the media and each sample was used separately for the next steps including assessment of colony forming potential or culturing for further analysis.

2. Preparation of single cell suspension from normal pulp

Human third molars were collected from 4 young adult patients after giving written informed consent and with the approval of the local Ethics Committee. The specimens were cut from around the root-enamel boundary using dental fissure burs. Pulp tissues were then gently removed from the chambers and digested as explained above. Stem cells from the pulp tissue were isolated according to previously-published methods (5). The cells were then resuspended in the media and each sample was used separately for the next steps including assessment of colony forming potential or culturing for further analysis.

3. Culturing the isolated cells

The single cell suspensions from both sources were plated in culture flasks within α-MEM supplemented with 4 mM GlutaMAX, 100 U/mL penicillin, 100 µg/mL streptomycin and 20% FBS (all from Gibco/ Invitrogen). The cells were cultured for 72 h at 37ºC in 5% CO2 and 90% humidity. Unattached cells and debris were then removed and fresh medium was added to the adherent cells. The medium was changed twice weekly until the flask reached 80% of confluency. Then the cells were released with trypsin–EDTA (Gibco/ Invitrogen) and sub-cultured. They were passaged three times before being used for differentiation or flow cytometric analysis.

4. Adipogenic differentiation

For adipogenic differentiation, the third passage cells from all the samples from both healthy dental pulps and pulp polyps were harvested as mentioned above and cultured in MesenCult medium supplemented with 10% Adipogenic Stimulatory Supplements (both from Stem Cell Technologies Inc, Vancouver, BC, Canada) at a density of 1.5×104 cells in a milliliter of medium within 2-chamber culture slides regarding the manufacturer’s guidelines. The cells were cultured for 3-4 weeks and half of the medium was exchanged only when the color of the media changed to yellow. As the cells showed appropriate morphological changes, they were analyzed with Oil-red O staining (Sigma). Briefly, to perform the staining, the cells were fixed in 4% formalin containing 1% calcium chloride for an hour. Afterwards, the cells were stained with Oil-red O solution for 10-15 minutes, counterstained with 70% ethanol for a minute, and washed with distilled water.

5. Osteogenic differentiation

For osteogenic differentiation, the third passage cells from all the samples from both healthy dental pulps and pulp polyps were harvested and then were cultured in NH-osteoDiff Medium (Miltenyi Biotec GmbH, Bergisch Gladbach, Germany) at a density of 3×104 cells in a milliliter of medium according to the manufacturer’s guidelines. The medium exchange was performed 2 times a week for 3 weeks. To approve the differentiation, after appropriate morphological changes, the cells were stained with Alizarin red (Sigma). Briefly, to perform the staining, the cells were washed once with PBS and fixed in methanol for 10 minutes. They were stained with the solution of 0.1M Alizarin Red in 25% Ammonia water for 24 hour and washed with distilled water.

6. Colony forming assay

To assess the efficiency of single cell derived colony formation (Colony Forming Unit Fibroblast assay [CFU-F]), primary cells from healthy pulps or pulp polyps were seeded into 6 well plates at a live cell concentration of 1000 cells in a milliliter of the above mentioned medium. Single cell derived colonies were defined as those units with more than 50 cells. The number of colonies was counted on the day before the colonies were merged, or as late as 14 days of culture.

7.Population Doubling Time analysis

To compare the growth characteristics of polyp derived cells and DPSCs, the third passage cells from cultures of both resources were seeded with the concentration of 2×104 cells in each well of 12-well culture plates. The cells from one of the wells were detached by Trypsin-EDTA and counted with hemocytometer every day. Based on the results, the growth curve was drawn and population doubling time was calculated during logarithmic growth phase using doubling time software (http://www.doubling-time.com).

8. Flow cytometry

To analyze the cell surface antigen expressions, the cells from the third passage were used. The isolated cells were incubated 10-30 minutes in dark environment with the following anti-human antibodies: CD90–Allophycocyanin (APC), CD34-FITC, CD56-Phycoerythrin (PE) and CD45-Peridinin chlorophyll protein (PerCP) (Miltenyi Biotec), CD14-FITC, CD166-PE, CD44-FITC, CD146-FITC, HLA-DR-PerCP, CD73-PE (BD Biosciences), CD105-PE (AbD Serotec, Kidlington, Oxford, UK) and STRO1-pure (With secondary anti-IgM FITC conjugated IgG, Santa Cruz Biotechnology Inc., Santa Cruz, CA, USA). Isotype-matched irrelevant monoclonal antibodies including Mouse IgG1-PE, IgG1-APC, IgG2a-FITC (AbD Serotec, Kidlington, Oxford, UK), IgG2a-PerCP, IgG2b-FITC and IgG2a-PE (Miltenyi Biotec) were used to exclude non specific staining of the cells. Flow cytometric analysis was performed on a FACS Calibur instrument (BD Biosciences) using the Cell quest as data acquisition software. The WinMDI 2.8 software was used for the data analyses.

9. Statistical analysis

Data were collected from the culture results and flow cytometry analysis. Mann-Whitney tests were used to show differences between results. P value < 0.05 was considered statistically significant. Graphpad Prism software (Graphpad, USA) was used for graphical presentation of data. 

## Results

1. Culture Characteristics

**Figure 1:**
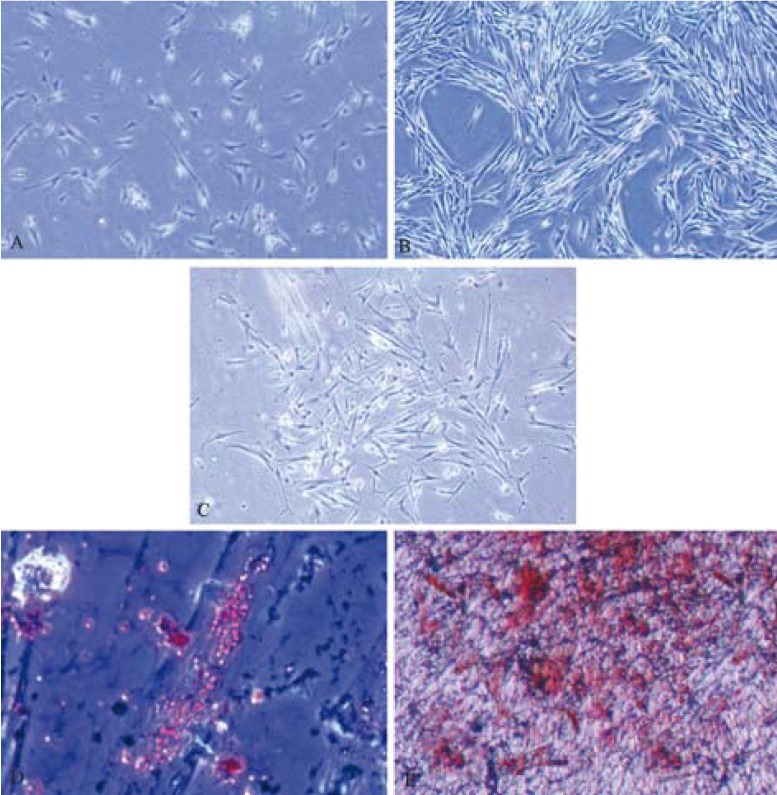
Pulp polyp derived cells. A) Five days after the initial seeding many cells were attached to the plates with appropriate fusiform like appearance. B) These fibroblast-like cells became confluent within 12-21 days. C) A single cell derived colony from pulp polyp derived cells formed within CFU-Fibroblast assay. D) Lipid vacuoles stained with oil red o stain after adipogenic differentiation. E) Osteogenic differentiation with calcium deposition identified by alizarin red staining. (*Abbreviations: CFU-F = Colony forming unit fibroblast*).

Two to five days after the initial seeding, pulp polyp derived cells were attached to the plates with appropriate fusiform like appearance (Fig. 1). These cells became confluent within 12-21 days with a typical fusiform fibroblast like appearance (Fig. 1). Furthermore, these cells could form ingle cell derived colonies successfully (Fig. 1). Cells from the normal pulp group showed the same morphology as mentioned above.

2. Differentiation assay

Adipogenic differentiation was confirmed through morphological changes and related staining. One week after seeding in the adipogenic medium, the cells showed small isolated vacuoles that increased in number and size with time and all of them were stained by Oil red O (Fig. 1). The earliest evidence of differentiation to osteoblasts was matrix depositions around the cells in the second week after seeding. Full differentiation to osteoblasts lasted for 4 weeks. Mineralization was documented by alizarin red staining (Fig. 1).

3. Colonogenic efficacy

**Figure 2:**
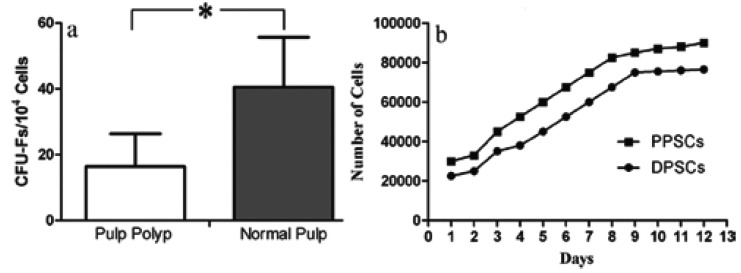
Comparison of characteristics from healthy pulp derived cells and polyp derived cells. A) Single cell derived clonogenic efficiency results. The colonies were counted 10 days after 104 cells were plated in the 6-well culture plates. B) Growth characteristics of pulp polyp stem cells (PPSCs) compared to DPSCs. Initially, 2×104 cells were plated in 12-well culture plates and the number of cells was counted each day. (Abbreviations: CFU-F =Colony forming unit fibroblast, DPSCs = Dental pulp stem cell, *P<0.05).

To assess single cell derived colony formation (Colony forming unit fibroblasts [CFU-F assay]), only colonies with more than 50 cells were considered in colony enumeration (Fig. 1). Normal pulp tissue derived cells formed higher colonies (40.5±15.1 among 104 cells) than the pulp polyp (16.3±9.9 among 104 cells, P = 0.016, Fig. 2).

4. Population Doubling Time analysis

The pulp polyp derived cells showed a mean of 3.86 days as a population doubling time. This result for DPSCs was 3.76 days (Fig. 2).

5. Flow cytometric results

**Figure 3:**
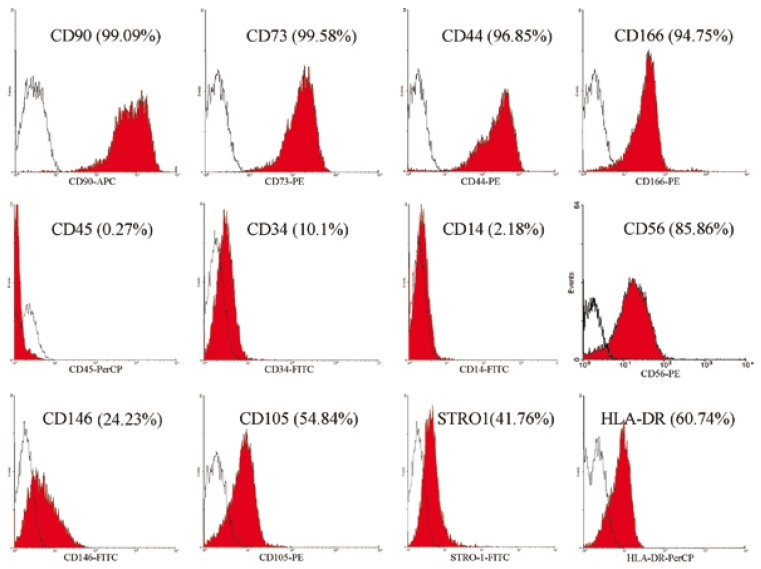
Flow cytometric analysis of PPSCs in vitro revealed a high level expression of CD44, CD166, CD90, and CD73 accompanied by low frequency for surface antigens: CD14, CD34 and CD45. But these cells show a heterogeneous phenotype for CD146, CD105, HLA-DR and STRO-1.

Flow cytometric analysis of the cells harvested from the pulp polyp derived cultures showed that the cells were positive for mesenchymal markers such as CD44, CD166, CD90 and CD73, and were negative for surface molecules CD14, CD34, and CD45. These cells show a heterogeneous phenotype for CD146, CD105, HLA-DR, STRO-1 and CD56 (Data from one of the samples is shown in Fig. 3). These results are comparable to those from normal pulp derived stem cells which were strongly positive for CD44, CD73, CD90, CD105, CD146 and CD166. DPSCs were negative for CD34, CD45, CD14 and HLA-DR, also showing heterogeneous phenotype for STRO1 and CD56. The Mean percentages of expression of all the markers from both resources are compared in  Table 1.

**Table 1:**
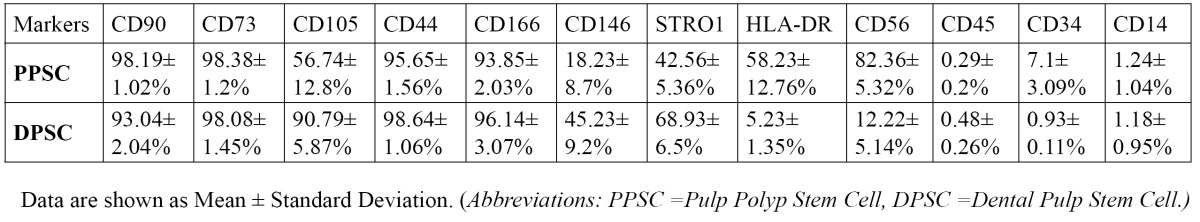
Percentage of cell surface antigens on the analyzed cells.

## Discussion

In the present study, we assessed the possibility of isolation of stem cells from pulp polyps and compared the characteristics of isolated cells with that of DPSCs. The cells isolated from the pulp polyp could successfully form CFU-F units with appropriate cell surface marker panel (expression of MSC markers: CD73, CD90, CD44, and CD166 in addition to the absence of hematopoietic markers: CD45, CD14, and CD34) and differentiation potential, all fulfilling the criteria from international society of cell therapy for MSC definition (1). From this perspective, we have considered these cells as stem cells and named them as “Pulp Polyp Stem Cells (PPSCs)”.

In our study, the normal pulp derived cells had a colonigenic efficacy of 40.5±15.1 among 104 cells. This is nearly similar to previous data in which colonigenic efficacy of DPSCs was shown to be a mean of approximately 50 CFU-Fs in 104 cells (14). Although the pulp polyp group showed a lower frequency of CFU-Fs among 104 cells than the normal pulp, it should be mentioned that generally polyp tissues are bigger in size than healthy pulps which leads to a higher number of total cells isolated from the enzymatically digested tissue. Eventually, the number of total CFU-Fs within a sample tissue, which can simply be estimated by multiplying the number of CFU-Fs among 104 cells by the total number of cells isolated from a tissue sample after enzymatic digestion, may be comparable to that from a healthy pulp.

DPSCs are the most commonly used stem cells isolated from dental tissue (6). They are commonly achieved when healthy teeth are extracted due to clinical implications. Few studies have been performed to assess the isolation of stem cells from pathologically affected dental tissues. Wang and colleagues have shown that the pulp affected by irreversible pulpitis, as a model of diseased pulp, contains viable cells with the potential for ex vivo expansion, proliferation and odonto-osteogenic differentiation capacity. These results suggest that even clinically compromised dental pulp tissues might have putative stem cells that could be used in endogenous pulp regeneration (11). The same is true regarding the inflamed peri-apical tissue (12) and inflamed periodontal ligament (15). Our study differs from the mentioned studies by the way that they evaluated the presence of stem cells in the normal tissues affected by inflammation, but pulp polyps are granulations tissues that are fully formed from a pathologic process. It has been shown that dental pulp stem cells activate as a result of odontoblastic injuries and the resultant inflammation prompts the proliferation and migration of stem cells (16). The migration of DPSCs explains their ability to detect and respond to tooth injury which ends in regeneration of the affected sites (17). It is demonstrated that the directional migration of DPSCs is mediated by both chemotactic factors and extracellular matrix proteins (18). Previous studies have identified that growth factors such as TGF-b1 might function as chemotactic gradients for DPSC migration after injury (19). Later, it was demonstrated that TGF-b1 was a less potent chemotactant than the blood-born sphingolipid mediator, sphingo-sine-1-phosphate (S1P). The other relatively weak chemotactants such as endothelial growth factor (EGFs) and fibroblast growth factor (FGFs) also have some potential to stimulate DPSC migration (18). The presence of stem cells in the pulp polyps may be attributed to stem cells proliferation and migration, but this presumption needs further basic cell biology research.

The use of PPSCs for clinical implications may have some limitations as well. Generally, the mesenchymal stem cells do not exhibit HLA-DR. However, BM-MSCs may up-regulate the expression of HLA-DR in inflammatory conditions while still not expressing hematopoietic markers such as CD14 and CD45 (20). Although such studies have not been performed on dental pulp stem cells, the high level expression of HLA-DR in PPSCs may have occurred as a result of chronic inflammation and follow the same order. This would reduce their suitability for allogenic transplantation purposes. Furthermore, pulp polyps are mainly exposed to oral cavity contents resulting in a higher possibility of contaminations. For this reason, we used anti-bacterial and anti-fungal supplementations since the moment of tissue collection to the final steps of sample preparation. As another disadvantage, it should be mentioned that there is a higher variability in the number of stem cells within a polyp than a normal pulp as the formation of the polyps follows a pathological process. In addition the frequency of stem cells within pulp polyps is lower than healthy pulp which can be demonstrated with formation of fewer CFU-Fs and lower expression of markers such as CD105, CD146, and STRO1. Finally, it should be noticed that polyps are rare tissues in daily practice and cannot be considered as a common finding in adult patients, so the clinical significance of PPSCs for autologous stem cell therapy may be limited.

Generally, according to the results of this comparative study, the pulp polyp as a diseased tissue contains appropriate amounts of stem cells with differentiation potentials comparable to those of the functional normal pulp. They are non-invasively acquired tissue resources usually discarded during endodontic therapies. This may provide a chance to access a new possible source of stem cells for affected patients.

## References

[1] Dominici M, Blanc KL, Mueller I Slaper-Cortenbach I, Marini F, Krause D, Deans R (2006). Minimal criteria for defining multipotent mesenchymal stromal cells. The International Society for Cellular Therapy position statement. Cytotherapy.

[2] Ahrari I, Attar A, Pourhabibi Zarandi N, Zakerinia M, Khosravi Maharlooei M, Monabati A (2013). CD271 enrichment does not help isolating mesenchymal stromal cells from G-CSF-Mobilized peripheral blood. Mol Biol.

[3] Ahrari I, Purhabibi Zarandi N, Khosravi Maharlooei M, Monabati A, Armin A, Ahrari S (2013). Adipose tissue derived multipotent mesenchymal stromal cells can be isolated using serum-free media. Iran Red Crescent Med J.

[4] Attar A, Langeroudi AG, Vassaghi A, Ahrari I, Maharlooei MK, Monabati A (2013). Role of CD271 enrichment in the isolation of mesenchymal stromal cells from umbilical cord blood. Cell Biol Int.

[5] Hadaegh Y, Niknam M, Attar A, Tavangar MS, Aarabi AM, Monabati A (2013). Characterization of stem cells from the pulp of unerupted third molar tooth. Indian J Dent Res.

[6] Huang J, Gronthos S, Shi S (2009). Mesenchymal Stem Cells Derived from Dental Tissues vs. Those from Other Sources: Their Biology and Role in Regenerative Medicine. J Dent Res.

[7] Miura M, Gronthos S, Zhao M, Lu B, Fisher LW, Robey PG (2003). SHED: stem cells from human exfoliated deciduous teeth. Proc Natl Acad Sci USA.

[8] Seo BM, Miura M, Gronthos S, Bartold PM, Batouli S, Brahim J (2004). Investigation of multipotent postnatal stem cells from human periodontal ligament. Lancet.

[9] Morsczeck C, Götz W, Schierholz J, Zeilhofer F, Kühn U, Möhl C (2005). Isolation of precursor cells (PCs) from human dental follicle of wisdom teeth. Matrix Biol.

[10] Sonoyama W, Liu Y, Yamaza T, Tuan RS, Wang S, Shi S (2008). Characterization of the apical papilla and its residing stem cells from human immature permanent teeth: a pilot study. J Endod.

[11] Wang Z, Pan J, Wright JT, Bencharit S, Zhang S, Everett ET (2010). Putative stem cells in human dental pulp with irreversible pulpitis: an exploratory study. J Endod.

[12] Liao J, Al Shahrani M, Al-Habib M, Tanaka T, Huang GT (2011). Cells isolated from inflamed periapical tissue express mesenchymal stem cell markers and are highly osteogenic. J Endod.

[13] Caliskan MK, Oztop F, Caliskan G (2003). Histological evaluation of teeth with hyperplastic pulpitis caused by trauma or caries: case reports. Int Endod J.

[14] Jo YY, Lee HJ, Kook SY (2007). Isolation and characterization of postnatal stem cells from human dental tissues. Tissue Eng.

[15] Park JC, Kim JM, Jung IH, Kim JC, Choi SH, Cho KS (2011). Isolation and characterization of human periodontal ligament (PDL) stem cells (PDLSCs) from the inflamed PDL tissue: in vitro and in vivo evaluations. J Clin Periodontol.

[16] Téclès O, Laurent P, Zygouritsas S, Burger AS, Camps J, Dejou J (2005). Activation of human dental pulp progenitor/stem cells in response to odontoblast injury. Arch Oral Biol.

[17] Magloire H, Romeas A, Melin M, Couble ML, Bleicher F, Farges JC (2001). Molecular regulation of odontoblast activity under dentin injury. Adv Dent Res.

[18] Howard C, Murray PE, Namerow KN (2010). Dental pulp stem cell migration. J Endod.

[19] Smith AJ, Murray PE, Sloan AJ, Matthews JB, Zhao S (2001). Trans-dentinal stimulation of tertiary dentinogenesis. Adv Dent Res.

[20] Shlomchik WD (2007). Graft-versus host disease. Nat Rev Immunol.

